# Infective Endocarditis in High-Income Countries

**DOI:** 10.3390/metabo12080682

**Published:** 2022-07-25

**Authors:** Francesco Nappi, Giorgia Martuscelli, Francesca Bellomo, Sanjeet Singh Avtaar Singh, Marc R. Moon

**Affiliations:** 1 Department of Cardiac Surgery, Centre Cardiologique du Nord, 93200 Saint-Denis, France; 2 Multidisciplinary Department of Medical-Surgical and Dental Specialties, University of Campania Luigi Vanvitelli, 81100 Naples, Italy; martuscelligiorgia@gmail.com; 3 Department of Clinical and Experimental Medicine, University of Messina, 98122 Messina, Italy; bellomofrancesca92@gmail.com; 4 Department of Cardiothoracic Surgery, Aberdeen Royal Infirmary, Aberdeen AB25 2ZN, UK; sanjeetsinghtoor@gmail.com; 5 Department of Cardiac Thoracic Surgery, Baylor College of Medicine, Texas Heart Institute, Houston, TX 77030, USA; moonm@bcm.edu

**Keywords:** infective endocarditis, *Staphylococcus aureus*, biofilm, immune response, fibronectin

## Abstract

Infective endocarditis remains an illness that carries a significant burden to healthcare resources. In recent times, there has been a shift from *Streptococcus* sp. to *Staphylococcus* sp. as the primary organism of interest. This has significant consequences, given the virulence of *Staphylococcus* and its propensity to form a biofilm, rendering non-surgical therapy ineffective. In addition, antibiotic resistance has affected treatment of this organism. The cohorts at most risk for Staphylococcal endocarditis are elderly patients with multiple comorbidities. The innovation of transcatheter technologies alongside other cardiac interventions such as implantable devices has contributed to the increased risk attributable to this cohort. We examined the pathophysiology of infective endocarditis carefully. Inter alia, the determinants of *Staphylococcus aureus* virulence, interaction with host immunity, as well as the discovery and emergence of a potential vaccine, were investigated. Furthermore, the potential role of prophylactic antibiotics during dental procedures was also evaluated. As rates of transcatheter device implantation increase, endocarditis is expected to increase, especially in this high-risk group. A high level of suspicion is needed alongside early initiation of therapy and referral to the heart team to improve outcomes.

## 1. Introduction

Infective endocarditis (IE) poses a significant challenge to health professionals which is currently greater than in the past. The reasons are manifold. The elderly, often with many comorbidities, who are the most affected patient population, have poorer reserves, and therefore become more unwell than previous cohorts [1,2,3]. Virulent staphylococci downgraded the role of *streptococci* and penicillin sensitivity (a hallmark of streptococcal infection), becoming the most common cause of IE in many high-income countries [4,5,6]. The population at risk of contracting IE has increased substantially alongside the risk of staphylococcal bacteremia during healthcare. This, today, represents the most important challenge in the world because it is the primary cause for the development of IE [7,8,9]. Increased resistance to many antibiotics is an alarming concern in modern-day healthcare because it constitutes a very serious threat [10,11,12]. 

There are some key points to consider. The first relates to a substantial discrepancy in the presentation of symptoms and in the course of the disease which does not avoid arousing concern for healthcare professionals [5,13]. One of the biggest challenges has been to create dedicated teams in which many specialists collaborate, including immunopathologists, microbiologists, infectious diseases specialists, cardiologists, cardiothoracic surgeons, and radiologists. The multispecialty team had to tackle the lack of evidence in international guidelines which are mainly based on observational cohort studies rather than on randomized studies regarding this subject [14,15,16]. Before the creation of multispecialty teams, people affected by IE were seldom assisted with high-level coordinated care, which persists as a problem in low-income countries where the assistance offered is often not meeting the level required to face the current challenge.

**Figure 1:**
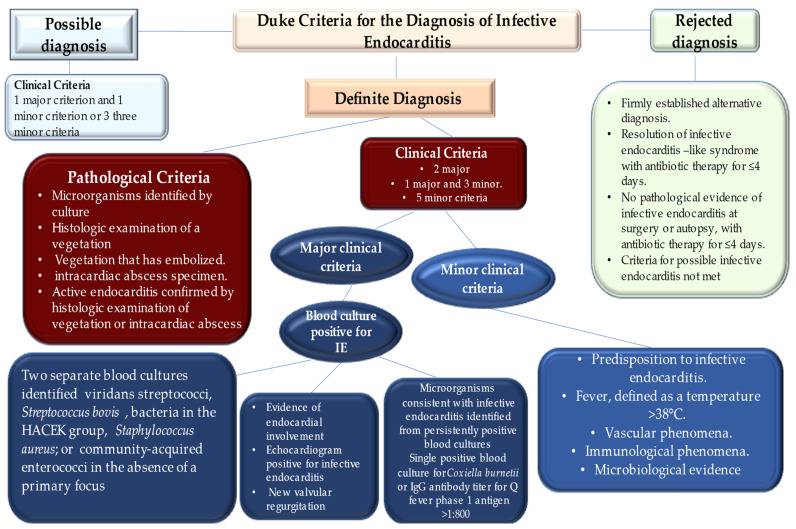
Depiction of Duke criteria for the diagnosis of infective endocarditis. HACEK indicates haemophilus species, *Aggregatibacter* (formerly *Actinobacillus*) *actinomycetemcomitans*, *Cardiobacterium hominis*, *Eikenella corrodens*, and *Kingella kingae*. For patients who have prosthetic valves and possible infective endocarditis according to clinical criteria, transoesophageal echocardiography is recommended. For patients with native valve endocarditis, transthoracic echocardiography is recommended.

The diagnosis of infective endocarditis is mostly reached by clinical, microbiological, and echocardiographic findings. The reliability of Duke criteria is critical, with sensitivity and specificity of more than 80%, and they remain the reference criteria for diagnosis [14,15,16]. It should be noted that during the diagnosis of IE, clinical judgment remains the priority choice for each individual patient, especially in the first phase of treatment, and it cannot be replaced by the Duke criteria (Figure 1).

As for clinical presentation, one of the main signs is fever, noted in up to 80% of patients with IE [1,2,3,4,5]. Individuals frequently experience, as reported in vast current case series, a new cardiac murmur or worsening of a known murmur in 48% and 20% of cases, respectively. Clinical investigations recognize other minor signs, such as hematuria in 25% of cases, splenomegaly in 11%, splinter hemorrhages in 8%, Janeway’s lesions in 5%, Roth’s spots in 5%, and conjunctival hemorrhage in 5% of cases. In such cases, clinical manifestations can be more severe and characterized by sepsis, meningitis, unexplained heart failure, septic pulmonary emboli, stroke, acute peripheral arterial occlusion, and renal failure, which may also be presenting manifestations [16,17,18,19,20]. Blood chemistry tests generally report the following changes in patients with IE: elevated inflammatory markers reveal a high erythrocyte sedimentation rate and C-reactive protein level in two-thirds of cases, while leukocytosis and anemia are found in about half of cases [1,21,22]. 

A total of 15 to 20% of patients disclose severe extracardiac complications of infective endocarditis that lead to cerebral damage [23,24]. The latter may include ischemic and hemorrhagic stroke, which crucially precedes the diagnosis of infective endocarditis in 60% of cases [25,26,27]. Again, other typical cerebral complications are silent cerebral embolism, transient ischemic attack, brain abscess, mycotic aneurysm, and meningitis.

A substantial concern is related to the specific characteristics of vegetations. These may be large, mobile, located on the mitral valve [26,27], and dependent on the infectious foci of the *S. aureus* infection [26,27,28,29,30,31] that have been linked with a notable augmentation of risk of symptomatic embolic events. A reliable diagnosis is offered by systematic magnetic resonance imaging of the brain that may highlight cerebral abnormalities in up to 80% of patients, including embolic events that occur asymptomatically in 50% of cases [32,33,34]. As for the events related to mycotic aneurysms, their development results from a septic arterial embolism which affects the intraluminal space or vasa vasorum. The second step is the spread of infection through the vessel wall. Mycotic aneurysms were recorded in 5% of older patients with IE [35,36], with detection more frequently registered recently because of the wider use of advanced imaging methods. Magnetic resonance angiography offers the best confirmation for depicting this lesion [37,38]. 

## 2. Special Populations of Infectious Endocarditis in the 21st Century

The major concern due to infective endocarditis is the discrepancy between the trends toward earlier diagnosis and surgical intervention with respect to the 1-year mortality that has not improved in over 2 decades. This indicates that infective endocarditis persists as a primary concern despite its change in presentation from the pre-antibiotic era, through the first generations of targeted antibiotic treatment, and finally to the present patient population who have all undergone variations in the microbiology profile [13,39]. In the past, IE occurred in young or middle-aged adults with underlying rheumatic heart disease or congenital heart disease (CHD). These patient populations have risk factors represented by prosthetic valve replacement, hemodialysis, venous catheters, immunosuppression, and intravenous (IV) drug use [40]. In the current era, the patient profiles include increasing age, frailty, and comorbidities which occur more frequently. At the same time, *Staphylococcus* strains became the most widely found causative pathogen, replacing oral streptococci [1,2,4,5]. 

Evidence suggests that, in the 21st century, the IE trend has seen an evolution that has led to the need for acquired healthcare in over 25% of cases [4]. Advances in cardiology have led to substantial changes in patient demographics and disease manifestation. Infective endocarditis greatly affects cardiac implantable electronic devices (CIED) [41,42]. The use of percutaneous catheter procedures for the treatment of structural heart disease may herald higher rates of infective endocarditis than those detected after prosthetic valve implantation performed with the standard surgical approach [42,43,44,45].

In this circumstance, it is of substantial importance to outline the epidemiology, pathophysiology, and pathological anatomy, to face the challenges posed by contemporary IE in developed countries. The analysis clarifies the reasons why diagnostics and therapeutic advances have failed to have a crucial impact on the disease.

## 3. Epidemiology

**Figure 2:**
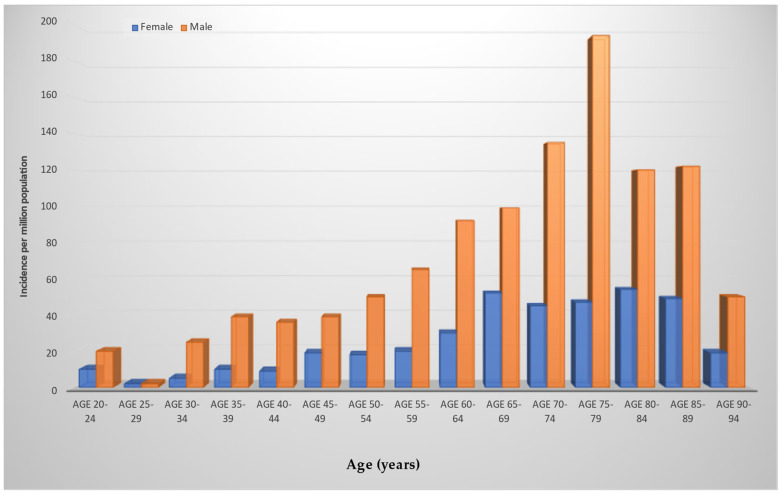
Depiction of the incidence of infective endocarditis according to age and sex. The incidence peak was reported as 194 cases per million in a population of men with IE aged 75–79 years. Blue box, female; dark yellow box, male.

Infective endocarditis seldom occurs, and its yearly incidence ranges between 3–10 per 100,000 individuals. Of note is that IE that occurs in persons who inject drugs (PWID) is rapidly increasing in the United States (US). Among this population, IE due to the use of heroin, which has established itself as the most common unlawful injected drug in the world, has almost doubled in the US between 2006 and 2013 to 681,000 active users [1,2,3,4,5,6,46,47] (Figure 2).

Variation in the pattern of IE has emerged worldwide with respect to the manifestation of disease during the early antibiotic era, whereas epidemiology in low-income countries was similar to that of high-income countries [4,5,6,48]. In low-income countries, the characteristic profile of rheumatic heart disease is revealed for two-thirds of cases as the principal key risk factor for infective endocarditis [49,50,51]. The disease occurs in young adult patients who develop the infection starting from oropharyngeal foci of penicillin-sensitive streptococci. Evidence of IE in high-income countries suggests a reduction in cases of rheumatic heart disease due to improved living standards and prophylactic administration of antibiotics to counter the spread of streptococcal pharyngitis [52,53]. A detailed evaluation of IE trends suggests that the main risk factors are represented by the increase in degenerative valve disease, diabetes, cancer, PWID, and congenital heart disease, which have replaced rheumatic heart disease. Furthermore, in analyzing the 2001–2006 epidemiological trend, infective endocarditis occurs at a higher rate in older individuals, between 65 and 70 years of age, compared to the average age in the early 1980s, which was reportedly around 40 years (Figure 1) [54,55].

The phenomenon of mutating epidemiology of infective endocarditis in high-income countries is related to substantial progress in the medical and surgical fields [56,57,58,59,60,61,62,63,64,65,66]. Therefore, an increase of 25–30% of contemporary cases of IE are acquired in the health sector, either due to the progressive increase in medical care offered during hospitalization or nosocomial admission, or to the possibility of contracting the infection on an outpatient basis [1,2,3,4,5,6,7]. In this context, there has been increasing use of long-term intravenous lines and invasive procedures, which constitute an ideal gateway for pathogens, leading to increased rates of staphylococcal bacteremia, which today represents the first precursor of infective endocarditis [67,68,69,70]. 

Advances in cardiological disciplines have allowed wider use of prosthetic heart valves and cardiac devices such as permanent pacemakers. The latter being in common use offers a higher risk of developing the infection inside the heart because it acts as a nidus for pathogens (Figure 1). Indications for implantation of complex devices such as cardiac resynchronization therapy and implantable cardioverter defibrillators have increased, consequently leading to an expansion of infection rates related to cardiac device implants [71,72,73,74].

**Figure 3:**
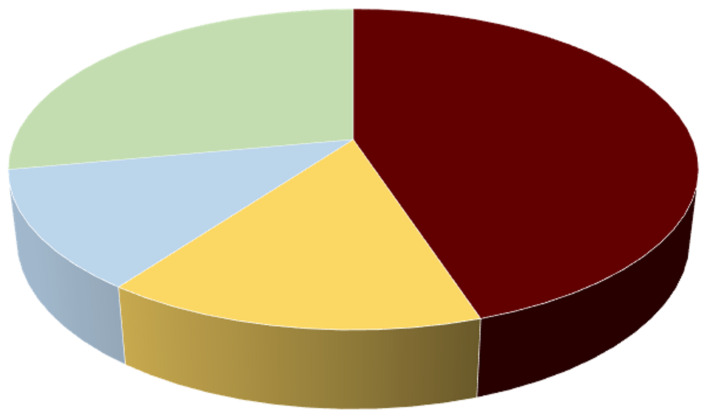
Depiction of the incidence of infective endocarditis according to the previous cardiac history in a French population study reporting data from 497 adults. Red dark box, no known cardiac disease; yellow box, prosthetic valve with or without intracardiac device; blue box, intracardiac device; green box, other cardiac device including individuals who received LVAD. Abbreviation; LVAD, left ventricular assist device.

The opposite spectrum is represented by the low incidence of infective endocarditis in the infant population. It is important to underline that, despite the evidence reporting a substantial improvement in survival in the population with congenital heart disease, which is the most important risk factor, an increase in the incidence of IE in recent decades has been recorded [75,76,77]. This increase mainly affects children with cyanotic congenital heart disease, high-velocity jets such as in cases of ventricular septal defect, or endocardial cushion defects [78,79,80]. There is a difference in the potential risk of contracting infective endocarditis which is due to specific conditions. A reduced incidence of IE is reported after performing a repair procedure without the appearance of a residual shunt or using autologous material compared to the prosthetic one. Conversely, the cumulative risk may be higher in patients who require more elaborate procedures to repair complex congenital heart diseases, valve diseases, residual shunts, or when prosthetic substitutes are used. For example, the incidence reaches up to 21% in 30 years after surgery for the surgical correction of aortic valve stenosis [81,82,83]. In pediatric patients without congenital heart disease, the cause of infective endocarditis is attributable to a complication arising from indwelling vascular catheters, as occurs in premature infants [84,85,86] (Figure 3).

## 4. Microbiology

A total of 80–90% of infective endocarditis is caused by the gram-positive cocci of the *Staphylococcus*, streptococcus, and enterococcus species. Among these pathogens, *S aureus* is the most frequently isolated causative bacterium associated with IE in high-income countries, causing up to 30% of infection cases [1,2,3,4,5,6,7,8,9]. Infective endocarditis determined by Staphylococcal foci affects several populations of individuals: those who are traditionally included in at-risk cohorts such as patients with hemodialysis treatment, in PWID, and others who can get the infection from native, prosthetic valves and cardiac implantable electronic devices (CIEDs) [41,72,73,74,87,88]. In addition, Cocci of the *Staphylococcus* manifest a deep-rooted propensity to acquire antibiotic resistance, thereby meticillin-resistant strains have emerged, constituting a grave concern worldwide [5,89,90].

The family of coagulase-negative staphylococci (CoNS), including *Staphylococcus epidermidis*, *Staphylococcus lugdunensis*, and *Staphylococcus capitis*, stands out as far-reaching skin commensals. Coagulase-negative staphylococci have specific characteristics. They frequently colonize indwelling lines, CIEDs, and are the most common causative pathogens found in patients suffering from early prosthetic valvular endocarditis [91,92,93,94]. They are often responsible for hospital-acquired native valvular endocarditis [95,96,97]. Furthermore, these commensals express the worrying function of producing biofilms, which can lead to high rates of abscess formation and promote multi-antibiotic resistance [97]. Babes et al. recently reported a 19.6% infection rate caused by *Staphylococcus aureus* leading to the development of large vegetations, prosthetic valve endocarditis, and intracardiac abscesses. CoNS coagulase-negative staphylococci occurred in 18.5% of infections leading to prosthetic valve dysfunction. In particular, the presence of *Streptococcus gallolyticus* etiology was correlated with ischemic embolic stroke and with the development of large vegetations [98].

In low-income countries, causative bacteria leading to infective endocarditis are streptococci, of which the oral viridans group persists as the most common causative germ [48,51]. Gram-positive Cocci of Streptococcus include *Streptococcus mutans*, *Streptococcus salivarius*, *Streptococcus anginosus*, *Streptococcus mitis*, and *Streptococcus sanguinis*, which are distinguished as commensals of the oral, gastrointestinal, and urogenital tract. Cases of infective endocarditis associated with underlying colon cancer have been found that were supported by group D streptococci (e.g., *Streptococcus gallolyticus*, *Streptococcus bovis* group, including only *Streptococcus equinus*), whose gateway was offered by the portal bloodstream. For the *Streptococcus bovis* group, the strains of *Streptococcus faecalis* and *Streptococcus faecium* were initially included in the serological group D. However, the latter two are separable from the other group D streptococci based on their ability to grow in 6.5 NaCl. %, hydrolyze arginine, and decarboxylate tyrosine; therefore, they were renamed enterococci. Infections linked to the development of enterococcal foci occur in 10% of individuals [1,2,3,4,5,6,7]. The type of germ isolated is mainly *Enterococcus faecalis*, which is the cause of both native and prosthetic valve endocarditis occurring in elderly or critically ill patients. Cases of IE sustained by *Enterococcus faecium* leading to increased resistance to vancomycin, aminoglycosides, and ampicillin have been reported [99].

**Figure 4:**
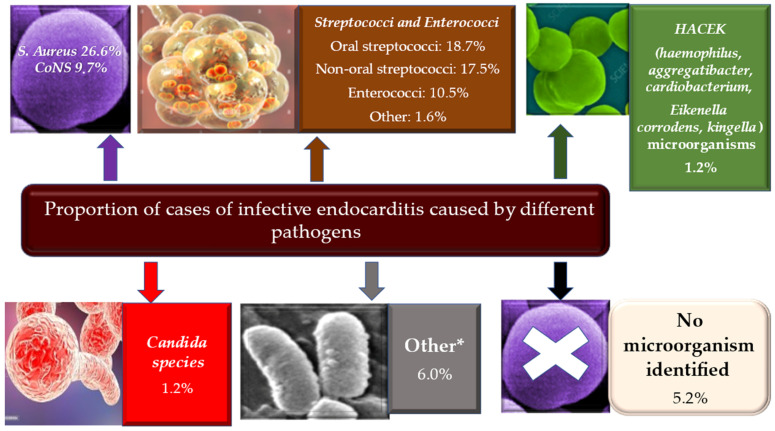
Major incidence of IE is revealed in elderly history of CIEDs and in younger population with history of PWID. Minor incidence in patients with central venous catheters, HIV, CHD, and immunosuppression. 26.6% of cases of IE are due to *Staphylococcus* aureus and CoNS are involved in 9.7% of cases * Low numbers of *Coxiella Burnetii*, *Bartonella quintana*, *Pseudomonas aeruginosa*, *Tropheryma whipplei*, *Enterobacteriaceae*, *Acinetobacter ursingii*, *Listeria monocytogenes*, *Propionibacterium acnes*, *Lactobacillus* spp., *Corynebacterium* spp, *Francisella tularensis*, *Erysipelothrix rhusiopathiae*, *Gordonia bronchialis*, *Bacillus* spp., *Catabacter hongkongensi*, *Moraxella catarrhalis*, *Campylobacter fetus*, *Neisseria elongata* and *Veillonella* spp. collected Abbreviations: CIED, cardiac implantable electronic devices; CHD, congenital heart disease; CoNS, coagulase negative; HIV; immunodeficiency virus; IE, infective endocarditis; PWID; persons who inject drugs.

From the conventional etiological model, it has emerged that a number of infectious endocarditis are mainly related to intracellular microorganisms, such as in cases of IE supported by causative pathogens including *C. burnetii*, *Bartonella* species or *Tropheryma whipplei*, in which host exposure and immune response may play a prominent role [99]. Therefore, about 10% of IE are represented by a mixture of fastidious bacteria, zoonotic bacteria, and fungi. Of particular interest are the HACEK bacteria colonizing the oropharynx (*Haemophilus*, *Aggregatibacter*, *Cardiobacterium*, *Eikenella corrodens*, *Kingella*), involving about 3% of individuals with IE. These pathogens are mainly characterized by slow growth [100]. Again, zoonotic endocarditis occurs after contact with pathogens such as *Coxiella burnetii* and *Brucella* (from cattle), *Bartonella henselae* (from cats), and *Chlamydia psittaci* (from parrots, pigeons). Lastly, rare pathogens include Gram-negative bacteria (e.g., *Acinetobacter* spp., *Pseudomonas aeruginosa*), *Legionella* spp., *Mycoplasma* spp. and *Tropheryma whippelii* [99,101]. Fungal infective endocarditis, usually caused by *Candida* or *Aspergillus*, are rare but often fatal causative germs, as well as the IE favored by *Histoplasma capsulatum*. These occur in patients who are immunosuppressed or who have had heart surgery, mostly with colonization affecting prosthetic valves. Recently, particular attention was given to *Borrelia* spp. as a causative pathogen that may be increasingly found as a cause of infective endocarditis. It should be highlighted that infectious disease specialists should keep in mind the likelihood of borrelial etiology of endocarditis in endemic areas [102,103] (Figure 4).

### 4.1. Biofilm Formation

**Figure 5:**
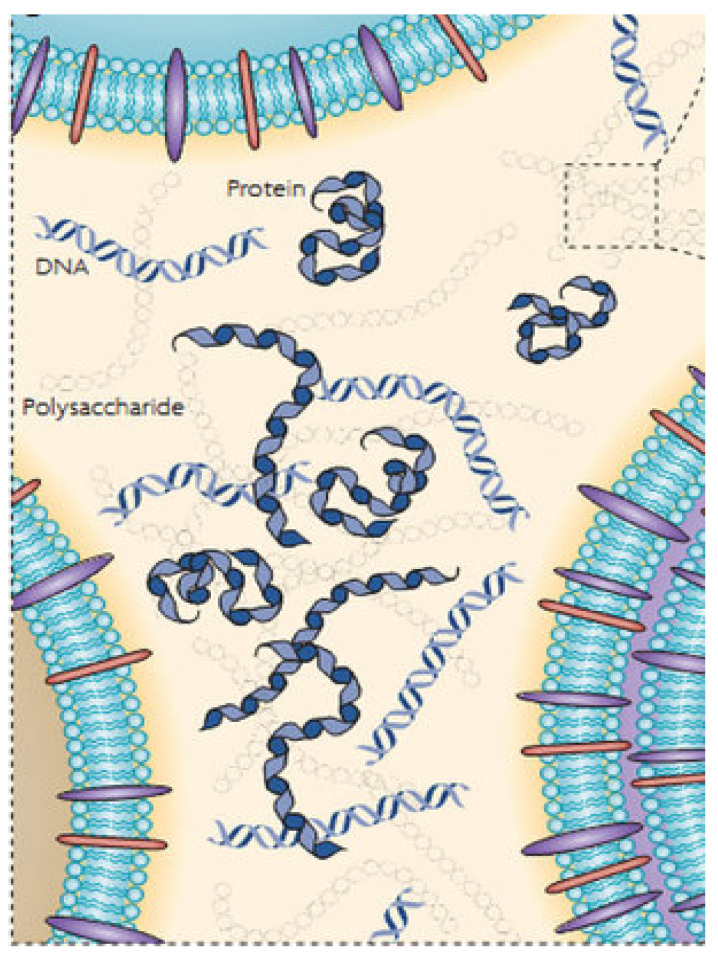
The main constituents that form EPS are molecules of polysaccharides, proteins, nucleic acids, and lipids. Abbreviation: EPS, extracellular polymeric substances.

In biofilms, microorganisms can live by adapting function and metabolism to a self-produced matrix which is made up of hydrated extracellular polymeric substances (EPS). This biofilm acts as an immediate functional environment formed directly by the bacteria. The main constituents that form EPS are molecules of polysaccharides, proteins, nucleic acids, and lipids (Figure 5).

EPS perform multiple functions, such as providing the mechanical stability of biofilms, mediating their adhesion to surfaces, and forming a cohesive, three-dimensional polymeric network that interconnects and transiently immobilizes the cells of the biofilm. It is important to underline the function of the external digestive system offered by the biofilm matrix. This step allows preservation of the extracellular enzymes close to the cells which can metabolize the dissolved, colloidal, and solid biopolymers [104,105,106,107].

During infective endocarditis, the formation of bacterial biofilms is a crucial moment for the nefarious evolution of the disease. IE begins as a minor injury of the heart structure and the damage generated is followed by a healing reaction, leading to the recruitment of fibrin and immune cells. In the first curative phase, the vegetations are sterile but potentially at risk of colonization during temporary bacteremia, thus leading to IE. Experimental in vitro models using simulated IE vegetation models produced from whole venous blood are of great use for the study of biofilms during infective endocarditis. In fact, these models make it possible to obtain stable bacterial colonization after 24 h. Once structured in biofilm aggregates, the pathogens show greater tolerance to antibiotics [106,107].

Swartz et al. recently evaluated the time to biofilm formation and how this impacts the development of antibiotic tolerance. Evidence suggests that reference strains of *Staphylococcus aureus*, as well as three clinical isolates of IE, formed biofilms on the IE vegetation model after 6 h. Furthermore, the earlier the antibiotic was administered, the more marked was its activity in containing the maturation of the biofilm, suggesting that early treatment was more effective in containing the development of the disease. The authors were able to follow the development of the biofilm under the microscope by viewing bacterial aggregates growing on the IE vegetation model and the interaction with the antibiotic. The formation of mature and antibiotic-tolerant biofilms was recorded after 6 h, thus accelerating screening for optimal treatment strategies for IE [108].

### 4.2. Staphylococcus aureus Protective Shield and Host Protection Mechanisms: New Evidence from Infectious Deployment

Several animal models of invasive *S. aureus* infections indicated two coagulases, von Willebrand factor-binding protein (vWbp) and Coagulase (Coa), as factors leading to its virulence. These proteins constitute a functionally intricate structure that *S. aureus* forms to create a protective shield formed of fibrinogen/fibrin surrounding it. The creation of this shield gives the microorganism the ability to evade the defense mechanisms exerted by the host’s phagocytic cells. One of the key functions of coagulases leads to the non-proteolytic activation of the zymogen prothrombin to convert fibrinogen into fibrin, thus promoting the formation of the fibrinogen/fibrin protective shield. 

**Figure 6:**
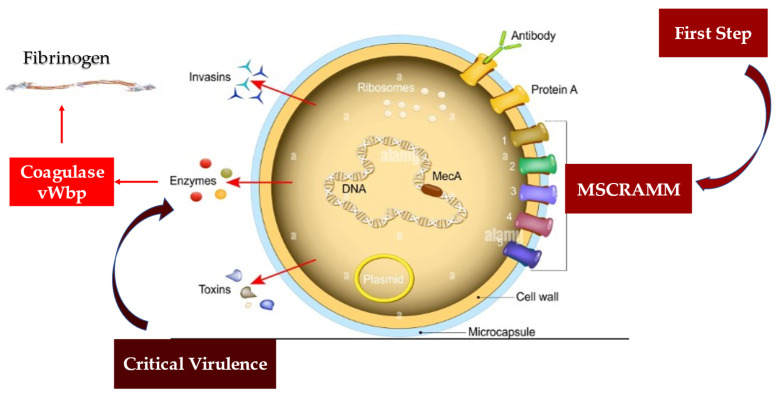
Depiction of virulent factors of *S. aureus.* MSCRAMMs have a substantial key role in driving the initiation of endovascular, bone and joint, and prosthetic device infections. These structures can bind to molecules such as collagen (mostly via Cna), fibronectin (via FnbAB), and fibrinogen (with ClfAB and Fib), and thus evade the immune system. The development of infection is induced by Coa and von Willebrand factor-binding protein that led to critical virulence. Coa binds preferentially to soluble fibrinogen, while vWbp does not disclose any preference between the two forms of fibrinogen. Abbreviations: Clf, cell-bound clumping factor; Coa, coagulase; Fnb, fibronectin binding protein; MSCRAMM, microbial surface components recognizing adhesive matrix molecules; vWbp, von Willebrand factor-binding protein.

Another characteristic offered by coagulases is their direct link with fibrinogen, whose interactions substantially support infection. The mechanism or mechanisms by which vWbp and Coa bind to fibrinogen involve distinct interactions of the two proteins with the molecule, despite their similar structure. The binding of Coa to soluble fibrinogen has a significantly greater affinity than fibrinogen coated on a plastic surface. On the other hand, the vWbp did not reveal any preference between the two forms of fibrinogen [109,110,111,112,113] (Figure 6).

The recent study by Thomas et al. provides crucial insights into the complex interactions between fibrinogen and *S. aureus* coagulase. The investigators suggest that vWbp and Coa target different sites on the fibrinogen, so there is no competition between the two molecules in fibrinogen binding. Both Coa and vWbp have N- and C-terminal halves that drive fibrinogen-binding activity [112,113].

Regarding the vWbp coagulase, the higher binding affinity for fibrinogen was identified in the vWbp-N region, contrary to Coa in which the greatest inclination toward the fibrinogen-binding site was expressed in the C-terminal region. Interestingly, it has been reported that the peptides constituting the previously identified Fibrinogen Coa/extracellular fibrinogen-binding motif (Efb1) fail to inhibit the vWbp-C component from binding to fibrinogen (Fg), suggesting for vWbp-C the absence of a functional homolog to this motif. Again, although the N-terminal prothrombin-binding domains of both coagulases recognized the fibrinogen β-chain, they appear to interact with different sequence motifs in the host protein. The interaction of the two coagulases seems to be expressed with different sequence motifs in the host protein. Collectively, our data provide insight into the complex interactions between Fg and the *S. aureus* coagulases [113].

Multidrug-resistant *S. aureus* strains cause life-threatening diseases and pose a worldwide public health problem. The limitations of dealing with staph infection depend on both the treatment and the lack of an effective vaccine. *S. aureus* develops complex and precise mechanisms that allow it to coat itself with a protective shield of fibrinogen/fibrin. This coating has two substantial effects: (1) it allows the pathogen to survive in the blood making it invisible to the host’s immune protection; and (2) it offers the possibility of spreading and causing invasive diseases. Modifying this process represents a promising goal for new antistaphylococcal treatment strategies; however, the mechanisms that characterize it are not yet fully elucidated. *S. aureus* expresses a number of proteins that bind to fibrinogen. A redundant action exerted by some of these proteins with vWbp can limit its function. In fact, in the case in which proteins express similar functions, a sharing between them in the structural or functional motif has often been suggested. Thomas et al. demonstrated the existence of a protein homologous (vhp) to the C-terminus of the von Willebrand factor-binding protein. This discovery makes a key contribution to both shield assembly and fibrinogen binding. Investigators identified a common Fg-binding motif between vhp and vWbp [113].

Recently, Schwartz et al. offered a very precise evaluation of the potential pathomechanisms involved in inducing infective endocarditis. The analysis was performed by studying 34 isolates of *S. aureus*, collected from patients with *S. aureus* endocarditis and from healthy individuals in both in vitro and in vivo models [114].

The strains of *S. aureus* isolated were tested in vitro to evaluate cytotoxicity, and the function of invading and interacting with platelets typically expressed by these pathogens. In order to correlate the ability of *S. aureus* to induce the development of vegetations on the aortic valves in vivo, the virulence factor expression profiles and cellular response were also studied and tested using an animal model. With the use of this method, the presence of IE involving valves was assessed by in vivo magnetic resonance imaging at 9.4 T, with histological evaluation and with enrichment gene expression analysis. *S. aureus* isolates were tested in vitro for their cytotoxicity, the potential for invasion, and interaction with platelets. All strains of *S. aureus* isolated and tested in vivo revealed the ability to cause IE and the inflammatory response associated with the aortic valve’s injuries; however, investigators were unable to differentiate and classify IE and inflammation based on the measurement of in vitro virulence profiles and cytotoxicity [114].

Of relevance, Schwartz et al. suggest that the in vitro test findings were unrelated to the severity of IE. However, a crucial finding highlights that the isolated *Staphylococcus* strains differed substantially in the degree of activation and inhibition of pathoanatomic processes related to the extracellular matrix and in the characteristic of the inflammatory response. Investigators, therefore, suggest that the pathogenic capacity of bacteria does not confer a uniform response, and comprehensive approaches to host–pathogen interactions are required for its evaluation. Furthermore, this approach offers the possibility to study the corresponding immune pathways in order to highlight the differences in the host–pathogen interaction [114].

Considering the etiology of *Staphylococcus aureus*-induced infective endocarditis, Schwarz et al. opened a window to reach a better understanding of the interaction between virulence factors and immune response in *S. aureus*-borne infective endocarditis, to offer new possibilities for the development of therapeutic strategies and specific diagnostic imaging markers [114].

## 5. Pathophysiology

**Figure 7:**
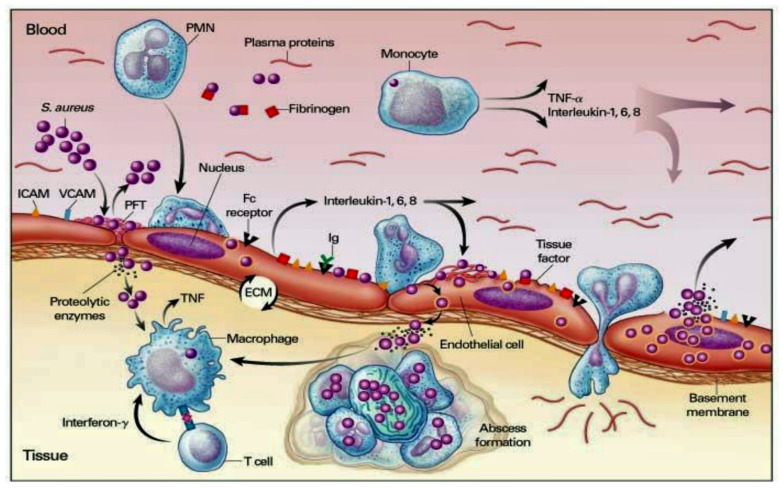
Bacterial adhesion induces the pathophysiological process of infective endocarditis. The first step led to inflammatory response with the involvement of inflammatory cells (PMN, monocyte, and macrophage). The inflammation is mediated by the production of cytokines (TNF, α, Il 1,6 and 8), integrins, tissue factor, and adhesion molecules (ICAM, VCAM), which in turn attract monocytes and platelets with associated production of fibronectin, due to the effect induced by chemokines. *S. Aureus* releases Cytoxins that trigger the immunity response both innate and mediate (T-cell and B-cell). Abbreviations: ICAM, Inter Cellular Adhesion Molecule; *S. Aureus*, *Staphylococcus aureus*; IL; interleukine; PMN, polymorphonuclear; TNF, tumor necrosis factor; VCAM, vascular cell adhesion molecule.

In the absence of cardiac pathology, the cardiac endothelium is not subject to the frequent bacteremia that can be induced by common daily activities, the most frequently represented by chewing and brushing the teeth [115]. Bacterial adhesion constitutes one of the fundamental stages in the pathophysiological process of infective endocarditis. Once the endothelial lesion is established, bacterial adhesion is favored, initially by the release of inflammatory cytokines associated with tissue factors and a second time by the expression of fibronectin, which leads to the formation of a thrombus composed of platelets and fibrin [116,117,118]. We learned that the common pathogens responsible for endocarditis colonize the valves with pre-existing sterile vegetations or valves in which minimal endothelial lesions occur. The inflammatory response established in the endothelium is orchestrated by the production of cytokines, integrins, and tissue factors, which in turn attract monocytes and platelets with associated production of fibronectin, due to the effect induced by chemokines. These structures allow the bacteria to attack and the latter further activate the inflammatory cascade which offers, through their incorporation, protection by the host’s defenses [117,118] (Figure 7).

There are three main injuries inflicted on the endothelium by an IE: valvular sclerosis, rheumatic valvulitis, or the direct activity of the bacterial pathogen. The latter is particularly induced by the intervention of *Staphylococcus aureus* in the infectious field [119]. The pathophysiological analysis of infective endocarditis starting from heterogeneous groups of individuals has ranges from those successfully treated without adverse events to subjects who suffered serious complications and high mortality. A change in the temporal trends of the IE model in high-income countries over the past 5 decades has resulted in changing pathophysiological mechanisms, involving increasingly unwell individuals who contract IE with increasing staphylococcal incidence and associated with healthcare. Consequently, based on pathophysiological knowledge, prevention strategies have adapted to the changing trend, with less use of prophylaxis against streptococcal bacteremia during dental procedures, and instead encouraging a more general approach to reduce the incidence of IE associated with healthcare. Therefore, practitioners acquire greater learning of the mechanisms of vegetation formation, growth, and embolization on damaged or inflamed heart valves and cardiac devices. A better understanding of these mechanisms has also led to increased knowledge of how to combat the growing problem of antimicrobial resistance. From a pathophysiological point of view, two mechanisms of IE have proved to be crucial in the treatment of IE: the role of the immune response in elderly patients with IE, and in particular after transcatheter implantation of the aortic valve, as well as the mechanisms that trigger septic shock. This latter condition leads to a substantial increase in the risk of death in patients with IE [120,121,122,123,124].

### 5.1. Staphylococcus aureus Immunity

#### 5.1.1. *Staphylococcus aureus* Interacts with Host Innate Immunity

*S. aureus* can have many virulence factors, both surface and secretory, which, once activated, offer a high capacity to oppose the host’s immune defense mechanisms [125,126]. The main virulence factor of *S. aureus* is the Accessory Gene Regulatory System (Agr), which works for pathogen quorum detection. Although we know that Agr works on controlling the expression of phenol-soluble modulins active against immune cells such as keratinocytes (KCs), how this mechanism is executed at the right time has not yet been demonstrated [127]. The innate immune response induces the response of dead KCs that produce a physical barrier due to the release of antimicrobial peptides such as human β-defensin 2 and 3, cathelicidins, and ribonuclease 7, which support bacteriostatic action against infection by *S. aureus*. It has been reported that the antibacterial function of KCs is also induced by pattern recognition receptors (PRRs) such as toll-like receptors (TLR) and nucleotide-binding oligomerization domain proteins. These two surveillance systems detect molecular patterns associated with invading pathogens, thus promoting timely defense against *S. aureus* [128,129]. The innate immune response is also supported by the action of other cells, such as dendritic, B and T, macrophages, mast cells, natural killers (NK), plasma cells, and fibroblasts in the dermis [130,131].

*Staphylococcus aureus* infection has been proven to be supported by several mechanisms by which violation of innate immune system triggers is established. Furthermore, the other two stages fuel the infection of the pathogen, through entry into the bloodstream and dissemination into the host tissue once it has left the bloodstream. These two phases are supported by the specific function of molecules expressed by *S. Aureus* which interact with the endothelium, the blood, and the extracellular matrix. First, FnBPA and FnBPB bind fibronectin and interact with integrin α5β1 on the surface of the vascular endothelium, thus triggering cell invasion and transmigration. The wallethic acid and lipoteichoic acid of *S. aureus*, polymers that form the bacterial envelope, intervene at this moment to promote the staphylococcal invasion of the host cells. In the second phase, staphylococci induce the formation of fibrin thrombi through the activation of the agglutination process mediated by Coa/vWbp and ClfA, and bind to vWF on the endothelial surfaces, generating the formation of polymers such as Ultra Large vWF (ULVWF). The third phase leads to the secretion of Hla by *S. Aureus*. Hla is a toxin that interacts with the ADAM10 receptor, favoring the cessation of the physiological barrier functions of the endothelium vascular system. Finally, the trojan horse model is activated, whereby neutrophils containing intracellular *S. aureus* embedded by phagocytosis peel off to inject bacteria into host tissues [130,131].

#### 5.1.2. Staphylo Cytotoxins Are a Trojan Horse for Excellent Immune Modulation

Since *S. Aureus* targets a wide variety of immune cells during infection, the pathogen’s release of cytotoxins is crucial. It releases leukocidins, hemolysins, and prostate-specific membrane antigen (PSM). Leukocidins include leukotoxins (Luk), such as LukED and LukAB, gamma hemolysin (Hlg) which includes HlgAB and HlgCB, and proviral load (PVL). Malachova revealed that LukAB is effective only on human polymorphonuclear leukocytes [132] and can kill dendritic cells, monocytes, and macrophages [133,134]. Recently, Alonzo reported that LukED recognizes C-C chemokine receptor 5 of the cellular receptor and induces the killing of dendritic cells, macrophages and lymphocytes [133,134]. On the other hand, at the micromolar level, a substantial role is played by PSM and human leukocyte antigen (Hla). The former acts with a noticeable ability to kill neutrophils after phagocytosis [135]. Furthermore, it can interact with disintegrin A and metalloprotease 1 (ADAM1), favoring the killing process of monocytes, macrophages, neutrophils, and T cells [136]. A fundamental point concerns the role of cytotoxins as a Trojan horse to promote the spread of *S. aureus*, which is distinct from the role offered by *S. aureus* in evading the host’s immune response. Cytotoxins govern by significantly dampening both the innate and adaptive immune responses, allowing protection of *S. aureus* during travel in the host [137].

#### 5.1.3. Loss of B-Cell vs. T-Cell Cooperation due to Cumulative Effects of B-Cell Deletion and Lack of T-Cell Help

The way in which *S. aureus* evades host immune surveillance is mediated by Staphylococcal Protein A (SpA) proteins, which are integrated into the architecture of the *S. aureus* wall. They are released during the growth of the pathogen. Silverman et al. and Goodyer et al. demonstrated the presence of five domains in the SpA which were involved in the binding of immunoglobulins. The five immunoglobulin-binding domains bind to the IgG Fcγ domain and the Fab domain of the VH3 IgG and IgM clan [138]. This activity is driven by the cross links of the B cell receptors which lead to the polyclonal proliferation of the B cells, thus favoring the activity of the superantigen SpA.

By studying the phases of the infection, different growth responses were observed evoking a variable expression of SpA. This event leads to the secretion of the Hla toxin which activates specific B lymphocytes in positions further away from *S. aureus*. This is the immunological reason that humans generally produce antibodies against Hla, despite most of the expressed SpA strains. It is important to consider that the Hla release function is also mediated from the cell wall of the pathogen.

**Figure 8:**
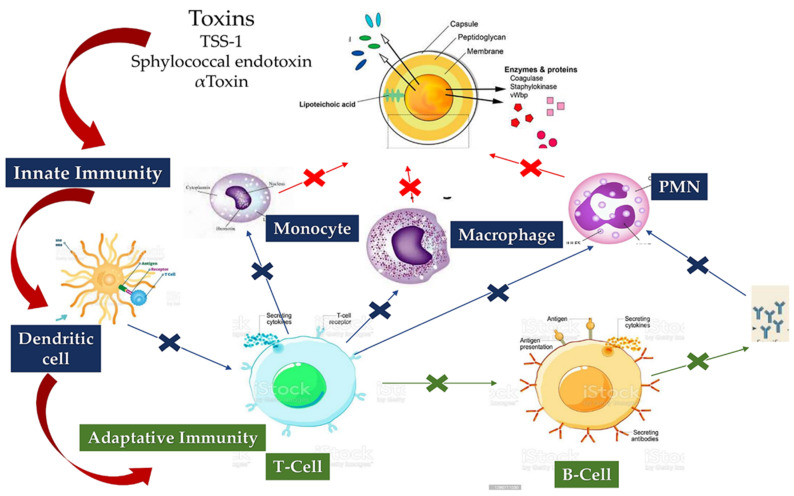
Staphylocytotoxins interfere (great blue arrow) with the cells of the innate (blue box) and adaptive (green box) immune response. Cytoxins (TSS-1, *Staphylococcal* endotoxin, and alpha toxin) are capable of lysing immune cells, including PMN, monocytes, and macrophages involved in the clearance of *S. aureus* (red arrow). Cytotoxins can also impair the function of adaptive immune cells (green arrows) represented by T and B lymphocytes. Finally, cytotoxins can impair the interaction between innate and adaptive immune cells (blue arrows). Abbreviation: TSS-1, Toxic Shock Syndrome-1.

Therefore, the superantigen activity exerted by SpA proteins can have an effect remotely from the infection, providing a crucial point for the development of the vaccine. A specific effect has been reported of SpA proteins which escape recognition by B cells resulting in a state called “lethargy”—a normal initial response to the antigen. In this case, the B cells may not collect a secondary signal to support their activation, leading to a state of shock called “anergy”. Anergy is a process that occurs in the colonization of *S. aureus*, in the persistence of its infection, and in the weakening of T cell help related to the fact that the effect of superantigens against T cells and cytotoxins leads to low affinity to the antibodies [139,140] (Figure 8).

#### 5.1.4. Immunoresponse and Vaccine

The development of a vaccine against the *Staphylococcus aureus* is an important goal related to the emergence of drug-resistant strains. The latter has resulted in driving investigations for alternative treatments, such as immunotherapeutic approaches. However, understanding the immune response to *S. aureus* infection and the production of an active vaccine go hand-in-hand. Whether the capacity of an *S. aureus* vaccine antigen can be extended to protect multiple mouse models infected with different strains of the pathogen has been reported in several published reports. This procedure allows the evaluation of immune cross-protection between different models in the presence of an unlikely strain of *S. Aureus.*

Concerns related to the development of effective humoral response may be mitigated by converging immunity-evading mechanisms of *S. aureus.* Unquestionably, the requirement for obtaining a promising vaccine in terms of efficacy and safety against *S. aureus* depends on a clear understanding of the immune, innate and adaptive, response. The immune response to *S. aureus* is articulate and comprises the humoral response, T cell help, blocking complement factors, and killing immune players by its toxins. All of these are the important determinants that require attention. The main contrasting mechanism exerted by *S. aureus* lies in hindering the immune action; this feature can lead to the failure of the development of targeted vaccines. Therefore, the crux of the matter may be the development of immunological interventions that can effectively obstruct the mechanisms by which *S. aureus* counteracts immunity. This process could ensure future success in vaccine development [141,142,143,144].

Early Secreted Antigenic Target 6 kDa-like proteins (ESAT-6) secreted by *S. aureus*, *S. aureus* EsxA (SaEsxA), and SaEsxB, as possible targets for a vaccine, were investigated. Although mice that were vaccinated with the administration of purified proteins elicited high titers of anti-SaEsxA and anti-SaEsxB antibodies, the immune response mediated by antibodies could not avoid *S. aureus* infection. On the other hand, mice treated with the use of recombinant SaEsxA (rSaEsxA) and rSaEsxB disclosed Th1- and Th17-biased immunity. In addition, they reported substantially improved survival rates when challenged with *S. aureus* compared with the controls. These results suggested that SaEsxA and SaEsxB functioned as two promising Th1 and Th17 candidate antigens, with potential expansion towards developing multivalent and serotype-independent vaccines against *S. aureus* infection [142].

Brady et al. worked on genetically inactivated alpha-toxin mutant HlaH35L and studies the protection afforded by this antigen in three models of infection using the same vaccine dose, regimen, route of immunization, adjuvant, and challenge strain. Using a systemic infection model challenged by mice immunized with HlaH35L, a limited but statistically significant decrease in bacterial colonization was recorded compared to that observed with control mice. Instead, using a prosthetic implant model of chronic biofilm infection, the investigators disclosed no significant differences in bacterial levels compared to controls. These findings suggest that, although vaccines may lead to protection against one form of *S. aureus* disease, they are nonetheless not active in offering protection against several disease manifestations and thus underline a significant challenge in *S. aureus* vaccine development [143].

The potential for *S. aureus* colonization reaches from 20 to 80% of humans, leading to a variety of illnesses constituting a real nightmare of healthcare- and community-associated bacterial infections [141,144]. In this context, vaccine development against *S. aureus* has failed, proving unsuccessful each time its application has been attempted to date. The reason is likely due to an insufficient comprehension of the mechanisms sustaining the immune defense against this pathogen. In humans, *S. aureus* provokes bacteremia, meningitis, endocarditis, pneumonia, osteomyelitis, sepsis, and skin and soft tissue infections. Individual carriers are at increased risk for infection and transmission to others. The spread of multidrug-resistant strains of *S. aureus* limits the optimal medical treatment with the use of antibiotic administration options [141,144].

Recently, awareness of opening a window on the future vaccine development against *S. aureus* strains provided notable insights. Zhang et al. illustrated the importance to generate multipronged B-cells, Th1-, and Th17-mediated, that effectively trigger a response against *S. aureus* antigens. Likewise, this precise immune response confers enhanced and broad protection against *S. aureus* and counteracts invasive infection, mucosal colonization, as well as skin and soft tissue infection [144].

Today, the impact of an immunotherapy approach is increasingly encouraged and supported, which in particular can be conferred by the administration of the vaccine against *S. aureus* infection. A crucial key role is played by the *S. aureus* manganese transport protein C (MntC). This protein is a highly conserved cell surface molecule that may arouse protective immunity against *S. aureus* and *Staphylococcus epidermidis*. Wei et al. studied the humoral immune response and CD4+ T cell-mediated immune responses, revealing essential protection for mice to reduce invasion of *S. aureus* evoked by MntC-specific antibodies. The evidence strongly reinforced the specific function of MntC-induced immunity response, revealing that Th17 played a remarkable part in preventing *S. aureus* infection. MntC-specific antibodies and MntC-specific Th17 cells act synergistically in preventing *S. aureus* infection. MntC-induced protective immunity decreased after neutralization of IL-17 by the antibody in vivo and adoptive Th17 transferred from mice may not be fully resistant to *S. aureus* [145].

### 5.2. Pathogen–Host Interaction in Determining Inflammation

Particular attention is paid to the substantial pathogenic action of *Staphylococcus aureus*, which is mediated by adhesion proteins, such as the fibronectin-binding protein and staphylococcal aggregation factors A and B, which play the role of bacterial mediators of adhesion and are the key determinants of pathogenicity [146,147,148,149]. Findings in an animal model with induced experimental endocarditis proved that the expression of *Staphylococcus aureus* adhesins in *Lactococcus lactis* play a crucial role of clumping factor A (ClfA) and fibronectin-binding protein A (FnBPA) for valve colonization [146].

Que et al. [146] evaluated the role of progression of infective endocarditis in animals that were followed for three days. The investigators noted that ClfA-positive lactococci successfully colonized damaged valves; nevertheless, the eradication of infection was spontaneously observed over 48 h. As for FnBPA-positive lactococci, pathogen titers progressively increased both in vegetations and in spleens. Imaging findings reveal that, while ClfA-positive lactococci were limited to the vegetations, FnBPA-positive lactococci also overran to the adjacent endothelium. This process explains the ability of FnBPA to trigger cell internalization in vitro. FnBPA carries both fibrinogen and fibronectin-binding domains, so the role of these two selective functionalities in causing infection was assessed by depriving FnBPA of the fibrinogen-binding domain and integrating it with the fibrinogen-binding domain of ClfA in cis. or in trans. Although the abrogation of the fibrinogen-binding domain of FnBPA did not change fibronectin binding and cellular internalization in vitro, it led to the complete elimination of valve infectivity in vivo. The ability to induce infection was restored in cis with the insertion of the fibrinogen-binding domain of ClfA into truncated FnBPA, while in trans was obtained by co-expressing full-length ClfA and truncated FnBPA, by using two separate plasmids. Therefore, it can be inferred that in *S. aureus* infection the binding of fibrinogen and fibronectin could cooperate for valve colonization and endothelial invasion in vivo [146].

*Staphylococcus aureus* infection is supported by bacteremia, which not only leads to complications such as infective endocarditis and osteomyelitis, but also promotes the pathogen’s exit from the bloodstream to cause metastatic abscesses. The bacterium’s interaction process with endothelial cells plays a substantial role in causing these complications. At this stage of the infection, several bacterial proteins have been shown to be involved. A fundamental role is offered by the extracellular adhesion protein of *S. aureus* (Eap), which has many functions, including binding various host glycoproteins [150,151,152,153,154].

It has also been shown to have both pro- and anti-inflammatory activity. Difficulties have emerged in robustly testing the role of Eap in vivo, due to the difficulties expressed in defining its activity in mutant strains. Substantial evidence has been reported on the pro-inflammatory role of Eap and on the activity that purified native adhesion protein of *S. aureus* has in triggering the release of TNFα in human whole blood in a dose-dependent manner. TNFα production promotes *S. aureus* adhesion to endothelial cells with a 4-fold increase, through a mechanism involving protein A on the bacterial surface and gC1qR/p33 on the surface of endothelial cells. This finding suggests that Eap’s contribution to disease severity during *S. aureus* bacteremia is crucial. It was genetically engineered for an isogenic set of strains, in which the Eap gene was inactivated and integrated after inserting an intact copy of the gene elsewhere on the bacterial chromosome. Using a mouse bacteremia model, it was shown that Eap-expressing strains cause a more severe infection, suggesting the major role of Eap in invasive disease [151,153,154].

**Figure 9:**
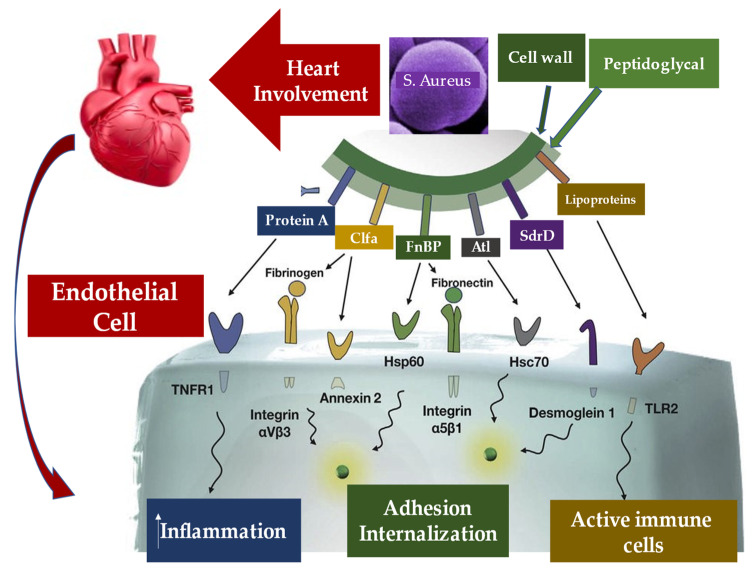
The substantial pathogenic action of *Staphylococcus Aureus* is depicted during infection of the heart and endothelial cells. The process involves three crucial stages: an increase in the inflammatory response (blue box), adhesion and internalization of the pathogen (green box), and the development of an active immune response (brown box). The external envelope of *S. Aureus*, consisting of the cell wall and peptidoglycans, expresses the different molecules involved in the three physopathological processes. Abbreviations: Atl, autolysin; Clfa, clumping factor A: FnBP, fibronectin binding protein; Hsc70, Heat shock cognate; Hsp60, Heat shock protein; SdrD, Serine Aspartate repeat containing protein D; TLR2, Toll-like receptor 2; TNFR1, tumor necrosis factor receptor 1.

Bacterial colonization offers the trigger for additional cycles of endothelial damage and thrombus deposition, resulting in the implantation of infected vegetation. In this phase, the production of a biofilm which is formed by a multilayer with a bacterial aggregate containing a polysaccharide associated with a protein matrix assists bacterial persistence and contributes to antibiotic tolerance [105] (Figure 9).

### 5.3. Interaction between Infective Endocarditis Pathogens, Vascular Endothelium, and Blood Constituents

Surface molecules of *Staphylococcus aureus* play a crucial role in the colonization of vascular endothelium, which is a fundamental primary event in the pathogenesis of infective endocarditis. The ability of these molecules to also launch endothelial procoagulant and proinflammatory responses, which lead to the development of IE, was extensively investigated [146,155,156,157,158]. Heying et al. [155] studied the individual abilities of three important molecules expressed on the *S. aureus* surface. Fibronectin-binding protein A (FnBPA) and B (FnBPA) and clumping factor A (ClfA) work to contribute to the bacterial adherence process that distinguishes the cultured human endothelial cells (ECs) when interacting with *Staphylococcus aureus*. Likewise, these molecules promote the phenotypic and functional changes in ECs. The method used included a non-invasive surrogate bacterium *Lactococcus lactis*, which, by gene transfer, expressed staphylococcal FnBPA, FnBPA or ClfA molecules. FnBPA- or FnBPB-positive recombinant lactococci lead to an increase of infection of ECs that reached 50- to 100-fold. Other important findings reveal EC activation, production of interleukin-8 associated with concomitant monocyte adhesion, as well as an augmentation of surface expression of ICAM-1 and VCAM-1. On the contrary, infections that were induced by ClfA-positive lactococci did not activate EC. The prominent action of FnBPA-positive *L. lactis* favored a significant response mediated by tissue factor-dependent endothelial coagulation that was enhanced by cell-bound monocytes. Evidence suggests that *S. aureus* FnBPs, but not ClfA, worked to invasiveness and pathogenicity to non-pathogenic *L. lactis* microorganisms, indicating that bacterium–EC interactions mediated by these adhesins were strongly prone to favor both inflammation and procoagulant activity at infected endovascular sites [155].

**Figure 10:**
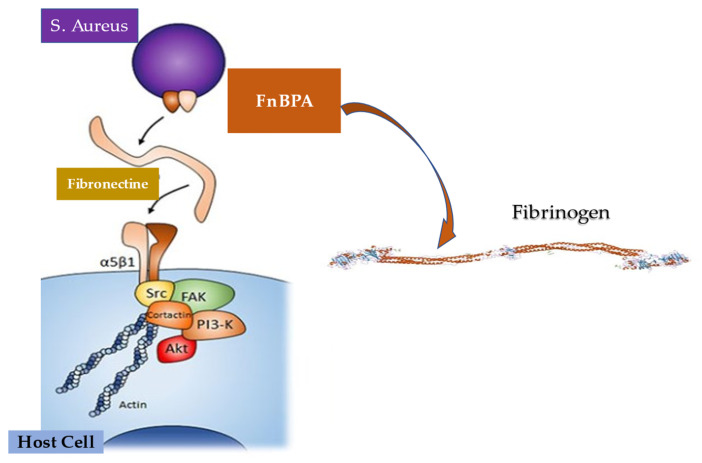
Experimental endocarditis induced by *S. aureus* marked the crucial function of sequential fibrinogen binding responsible for valve colonization and the paramount action of fibronectin binding leading to endothelial invasion. FnBPA responsible for fibrinogen and fibronectin binding may promote cell invasion in vivo endocarditis.

Experimental endocarditis induced by *Staphylococcus aureus* experimental endocarditis anticipated the function of sequential fibrinogen binding responsible for valve colonization and the paramount action of fibronectin binding that leads to endothelial invasion. These processes are sustained by peptidoglycan-attached adhesins. The function exerted by fibronectin-binding protein A favored a synthesis between these two specific properties, combined with the binding of elastin, in promoting experimental endocarditis. Piroth et al. revealed the minimal subdomain of FnBPA responsible for fibrinogen and fibronectin-binding may promote cell invasion in vivo endocarditis [156]. FnBPA was expressed in *Lactococcus lactis* and tested in vitro and in animals. The subdomain needed in determining infective endocarditis consisted of 127 amino acids, which represented the fulcrum of the FnBPA fibrinogen-binding and fibronectin-binding regions and were sufficient to confer the charge of these properties. Although evidence in animals supports the substantial role of fibrinogen binding to lead endocarditis induction, the work of fibronectin binding was not significantly associated with endocarditis induction. On the contrary, as for disease severity, both fibrinogen binding and fibronectin binding were crucial. In addition, the synergic combination of fibrinogen binding and fibronectin binding suggest a remarkable increase in the infectious invasion of cultured cell lines, underlining a critical characteristic to be correlated with endocarditis severity. As a consequence, the idea of a sequential action of fibrinogen binding and fibronectin binding in promoting colonization and invasion fell in support of the unexpectedly intertwined role offered in endocarditis by fibrinogen binding and fibronectin binding in terms of both functional anatomy and pathogenetic mechanism. This refined and unexpected feature of FnBPA paves the way for the development of anti-adhesin strategies [156] (Figure 10).

Microbiologists learned that bacterial proteins such ClfA and FnBPA intervene to mediate adhesion to endothelial cell (EC) surface molecules. This function is associated with the role of subendothelial matrix proteins, including fibrinogen, fibrin, fibronectin, and vWF [157]. Again, Pappelbaum et al. reported that ULVWF substantially contributed to the initial pathogenic step of *S. aureus*-induced endocarditis in patients who disclosed an intact endothelium. The use of heparin and A disintegrin and metalloprotease with thrombospondin type 1 repeats (ADAMTS13) to reduce ULVWF production may suggest the use of novel therapeutic options to prevent infective endocarditis [158].

Three reports recently investigated the synergistic role of ClfA, FnBPA, and von Willebrand factor in determining the adhesion of *S. aureus* to ECs and markedly confirm the fundamental importance of these molecules in IE [159,160,161]. 

In a first, recently published report, Claes et al. explained by demonstrating that vWbp interacts with a staphylococcal surface protein, mediating *S. aureus* adhesion to VWF and vascular endothelium under shear stress. The method used included various Sortase A (SrtA)-deficient mutants and SrtA-dependent surface proteins, as well as *Lactococcus lactis* expressing single staphylococcal surface proteins. The authors suggest that *S. aureus* first bound to the endothelium via VWF, subsequently secreted VWF-binding protein (vWbp), mediated the adhesion of *S. aureus* to VWF under shear stress, and finally, vWbp interacted with VWF and the Sortase A ClfA-dependent surface protein. Therefore, VWF–vWbp–ClfA anchored *S. aureus* to the vascular endothelium under shear stress [159].

In another publication, Claes et al. examined the influence of shear flow and plasma on the binding of ClfA and FnBPA, including its sub-domains A, A16+, ABC, CD, vWF, fibrinogen /fibrin, fibronectin or confluent ECs. The method used a genetically engineered *L. lactis* that expressed these adhesins heterologously. The investigators revealed that global adherence profiles were similar in static and flow conditions. Notably, in the absence of plasma, *L. lactis*–ClfA binding to fibrinogen increased with shear forces, whereas binding to fibrin did not produce the same effect [160].

The degree of adhesion of *L. lactis*–FnBPA to EC-bound fibronectin and of *L. lactis*–ClfA to EC-bound fibrinogen was similar to that of *L. lactis*–ClfA to coated vWF domain A1, in the presence of the vWF-binding protein (vWbp). Interestingly, in plasma, the adhesion of *L. lactis*–ClfA to activated EC–vWF/vWbp decreased by 80% in 10 min and was related to disintegrin-mediated and metalloproteinase-mediated vWF hydrolysis with thrombospondin motif type 1, member 13. Likewise, in absence of plasma, the adhesion of *L. lactis*–FnBPA was reduced by >70%, comparatively. In contrast, plasma fibrinogen supported high *L. lactis*–ClfA binding to resting and activated ECs. The investigators offered the explanation that, in plasma, *S. aureus* adhesion to active endothelium occurs mainly via two complementary pathways: a rapid but short-lived vWF/vWbp pathway and a stable integrin-coupled–fibrinogen pathway. In consequence, these results support the pharmacological inhibition of ClfA–fibrinogen interactions, which may constitute a valuable additional treatment in infective endocarditis [160].

The detrimental action caused by *Staphylococcus aureus*, which actively invades the endothelium, induces apoptosis and endothelial damage. We know that the role of *S. aureus* is crucial in causing IE because it promotes infection through the key role offered by protein-clotting factor A, which is associated with the cell wall of *S. aureus.* On the other hand, the role played by secreted plasma coagulation factors Staphylo-coagulase and by the protein binding von Willebrand factor has recently been clarified. Mancini et al. [161] described, in rats with catheter-induced aortic vegetations, the role of staphylococcal-secreted coagulase (Coa-positive staphylococci) and *S. aureus* encodes a von Willebrand factor-binding protein in the initiation of infective endocarditis. They used *Lactococcus lactis* mutants expressing coa, vWbp, ClfA or vWbp/clfA and *S. aureus* Newman Δcoa, ΔvWbp, ΔclfA or Δcoa/ΔvWbp/ΔclfA. The researchers noted that vWbp expression statistically increased *L. lactis*-induced valve infection compared with parental and Coa-expressing strains. Similarly, the expression of ClfA revealed increased infectivity of *L. lactis*, which was not further affected by the co-expression of vWbp. Importantly, deletion of the Coa or vWbp genes in *S. aureus* did not reduce infectivity, while deletion of ClfA dramatically reduced valve infection. Importantly, the activity of ClfA was not affected by the triple deletion of Δcoa/ΔvWbp/ΔclfA. Evidence has suggested that Coa does not support initial IE colonization by using *L. lactis* as the pathogen without other key virulence factors. Unquestionably, the presence of vWbp contributes to the onset of IE induced by *L. lactis*, but its role is marginal in the presence of ClfA [161].

We learned that *Staphylococcus aureus* has generally been contemplated as an extracellular pathogen; however, these microorganisms have also the ability to be integrated by host cells, including certain phagocytes. Hence, they may work inside endothelial cells, epithelial cells, or osteoblasts. The intracellular *S. aureus* position concurs with the establishment of infection. The entry gate of pathogens is mediated by the binding of integrin α5β1 expressed on the membrane of the host cell, which recognizes fibronectin. This bridge facilitates the recognition between pathogen and host cell, leading to subsequent cell integration [162,163,164,165]. Although the osteoblasts evidenced high expression of α5β1-integrin and fibronectin, and the bacteria disclosed a high affinity to adhere to osteoblasts, Niemann et al. demonstrated, through internalization tests and immunofluorescence microscopy, that *S. aureus* was less swallowed in osteoblasts compared to epithelial cells [166].

During cell infection with *S. aureus*, the authors added exogenous fibronectin, which resulted in increased uptake in epithelial cells that was not recorded in osteoblasts. This finding supports a clear contrast to previous claims regarding the pathogen uptake mechanism, which gave integrin and fibronectin expression a key role in promoting bacterial uptake in host cells. The organization of extracellular fibronectin surrounding osteoblasts and epithelial cells is different. In the former, it is organized in a fibrillar network. The investigators reported a significant increase in osteoblast uptake of *S. aureus*, resulting in inhibition of fibril formation, brief reduction in RNA-mediated fibronectin expression, and disruption of the fibronectin–fibril network. From the work of Nieman et al. it emerges that the fibronectin–fibril network appears to strongly reduce the absorption of *S. aureus* in a given host cell, indicating that the supramolecular structure of the fibronectin can direct the different ability of particular host cells to internalize the pathogen [166].

The recent study by Niemann et al. suggests the non-determining role played by the crude quantity of fibronectin, but rather the substantial role established by the supramolecular structure of the fibronectin molecules. Once deposited on the eukaryotic cell surface, they play an essential role in bacterial uptake by host cells. These results can explain the great variability expressed in the efficacy of *S. aureus* absorption, considering different types of host cells. Again, differences were found in vivo between the courses of bacterial infections and the localization of bacteria in different clinical settings [166].

The molecular basis of the pathogenicity of *S. aureus* is related to the expression of a variety of virulence factors, including proteins that mediate adherence to the host plasma and extracellular matrix proteins. Between these, evidence proved that IsdB-expressing bacteria bound to both soluble and immobilized vWF [167]. More recently, Alfeo et al. discovered that the iron-regulated surface determinant B (IsdB) protein, besides being involved in iron transport and vitronectin binding, interacts with vWF [168].

The researchers found that the bond established between IsdB and the recombinant vWF was stopped by heparin and was reduced due to high ionic strength. Furthermore, using the administration of ristocetin, an allosteric agent that promotes exposure to the A1 domain of vWF, the substantial effect of enhancing the binding between IsdB and vWF was obtained. An important finding supports that IsdB binding and *S. aureus* adhesion were significantly inhibited by a monoclonal antibody against the A1 domain, as well as IsdB-reactive IgG isolated from patients experiencing staphylococcal endocarditis. Therefore, the reported evidence suggests both the importance of IsdB in favoring the adhesion of *S. aureus*, and its role in the colonization of the endothelium by *S. aureus*. IsdB can serve as a potential therapeutic target [168].

### 5.4. Infective Endocarditis and Platelets

Although the use of antibiotic prophylaxis is presently recommended in patients with high-risk infective endocarditis, infective endocarditis persists with the features of a challenging disease and statistically confirms its higher mortality. Furthermore, the concerns related to the administration of antibiotics are confronted with their low efficacy, further contributing to the emerging infection rate for the selection of antibiotic-resistant strains. Given this scenario, the need to find new therapeutic strategies remains a firm point against IE. Platelets are essential in the initial phase of infective endocarditis, acting as first-line immune responders [148,149,169].

Evidence based on in-vitro mechanistic studies shows that the work undertaken by platelets is of crucial importance in the initial phase of infective endocarditis, constituting the first front of the immune response. The first phase of the disease is supported by the interaction of pathogens with platelets, for which counteracting platelet antimicrobial activity is a priority. Experimental in vitro and animal models have suggested that the effect of aspirin can limit bacterial–platelet interactions leading to the prevention of vegetation development and showing promising results. However, the data evoked in clinical studies on the outcome of patients with infective endocarditis who undergo medical therapy with aspirin administration remain controversial. Conflicting results cast a veil of uncertainty about the benefit of antiplatelet agents in the prevention of infective endocarditis. In the same way, in addition to aspirin, a therapeutic effect has been attributed to the antagonist of the platelet receptor P2Y12, ticagrelor, which would combine its powerful and well-known antiplatelet activity with strong antibacterial properties. Furthermore, a recent study based on a mouse animal model reported a marked ability of ticagrelor to eradicate *Staphylococcus aureus* bacteremia [169,170,171].

## 6. Evidence from Deploying Maneuvers as a Risk Factor for Bacteremia Related to Infective Endocarditis

Although *Staphylococcus aureus* remains the undisputed leading causative pathogen in infectious endocarditis, attention must be paid to those microorganisms such as *Porphyromonas gingivalis*, *Aggregatibacter actinomycetemcomitans* and *Streptococcus mutans* that mainly occur as aetiologic agents of dental caries and aggressive periodontitis. These bacteria can pose concerns for populations at risk of infective endocarditis [172,173,174,175,176,177,178].

### 6.1. Special Population Requiring Attention

#### 6.1.1. Tooth Extraction and Tooth Brushing

Lockart et al. compared the incidence, duration, nature, and magnitude of endocarditis-related bacteremia in patients who had single-tooth extractions and toothbrushing [115,176]. The authors determined the impact of amoxicillin prophylaxis on single-tooth extraction. A total of 290 individuals were enrolled for randomization in a double-blind, placebo-controlled study, as follows: (1) toothbrushing, (2) single-tooth extraction with amoxicillin prophylaxis, or (3) single-tooth extraction with an identical placebo. Blood was drawn for bacterial culturing and identification at six time points before, during, and after these interventions. The investigators focused their analysis on the role of bacterial species that was reported to lead to infective endocarditis. A total of 98 bacterial species were identified, and 32 of these were revealed to be the cause of endocarditis. Results suggest that cumulative incidence of endocarditis-related bacteria from all siz blood draws was detected in 23%, 33%, and 60% of the toothbrushing, extraction–amoxicillin, and extraction–placebo groups, respectively (*p* < 0.0001). Interestingly, the prophylaxis administration of amoxicillin resulted in a significant decrease in positive cultures (*p* < 0.0001). The findings of Lockhart’s study suggest that, although amoxicillin has a significant impact on bacteremia resulting from single-tooth extraction, as the increased frequency of oral hygiene is exerted by tooth brushing, the latter may represent a greater threat to people at risk of infective endocarditis [115].

The landmark randomized clinical trial of Lockhart et al. substantially supports that oral hygiene and gingival disease indexes are associated significantly with the development of infective endocarditis-related bacteremia after toothbrushing. Individuals enrolled with a mean plaque and calculus scores of 2 or greater revealed an increased risk of developing bacteremia between 3.78- and 4.43-fold. The investigators found that the occurrence of generalized bleeding after toothbrushing was associated with an almost eightfold increase in risk of developing bacteremia. However, no remarkable link was reported between any of the estimates of periodontal disease and the incidence of bacteremia after tooth brushing. Interestingly, Lockart et al. found that the oral hygiene or disease status of a tooth was not crucially related to the manifestation of bacteremia after dental extraction [176].

The manifestation of IE in the young population of patients requiring cardiac surgery has aroused great interest. A double-blind, randomized, placebo-controlled study evaluated the impact of amoxicillin prophylaxis on the incidence, nature, and duration of bacteremia from nasotracheal intubation and dental procedures in children, as well as for the impact of antibiotic prophylaxis on the incidence, nature, and duration of bacteremia in individuals after intubation and dental procedures. Lockart et al. reported that at 1.5 min after the initiation of dental extractions, bacteremia occurred in 76% of the children enrolled in a placebo cohort, compared to 15% of the amoxicillin group (*p* < 0.001). Evidence suggests that bacteremia occurrence rates were higher in the placebo group of children who received specific treatment as intubation, after dental restorations and cleaning, compared to those who were managed with amoxicillin (18% and 20% vs. 4% and 6%; *p* = 0.05 and *p* = 0.07, respectively). It is important to note that, in the majority of the 152 positive cultures and of the 29 different bacteria, the causative pathogens responsible for IE were Gram-positive cocci. Individuals included in the placebo group disclosed bacteremia that persisted longer over time [177].

#### 6.1.2. Causative Pathogens of Interest and Related Mechanism Leading to Disease

**Figure 11:**
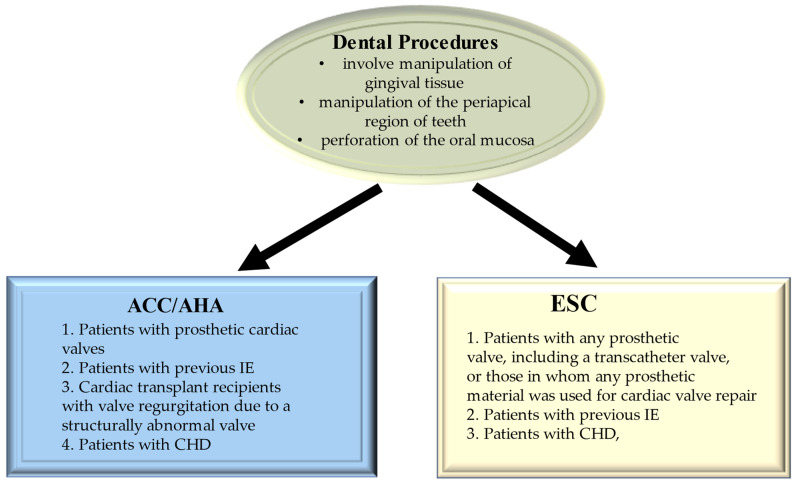
2020 ACC/AHA and 2021 ESC Guidelines on Use of Antibiotic Prophylaxis for the Prevention of Infection of Heart Structure. Abbreviations: ACC; American College of Cardiology; AHA, American Heart Association; ESC, European Society of Cardiology.

*Porphyromonas gingivalis* is considered a major periodonto-pathogen and is responsible for the pathogenesis of periodontitis. This process is mediated by increased production of Interleukin-1β (IL-1β), which work at regulating innate immune responses with a crucial function excreted in the host’s defense against bacterial infection. However, evidence proves that an excessive IL-1β is related to periodontal demolition. Again, TLR signaling and inflammasome activation substantially influenced IL-1β synthesis, maturation, and secretion, with higher levels of inflammasome components in the gingival tissues of patients with chronic periodontitis than in those from healthy controls. Park et al. investigated the molecular mechanisms by which *P. gingivalis* infection causes IL-1β secretion, focusing the findings on the characteristics of *P. gingivalis*-induced signaling in differentiated THP-1 cells. Importantly, the activation of TLR2 and TLR4 anticipated *P. gingivalis*-induced IL-1β release. *P. gingivalis* infection evoked a higher secretion of IL-1β associated with inflammatory cell death via caspase-1 activation. Both increased IL-1β secretion and pyroptotic cell death were sustained by NOD-like receptor (NLR) family, pyrin domain-containing 3 (NLRP3), and interferon-inducible protein AIM2 (AIM2) inflammasome activation. The activation of the NLRP3 inflammasome was mediated by ATP release, the P2X7 receptor, and lysosomal damage. The innate immune response against *P. gingivalis* infection which could potentially be used for the prevention and therapy of periodontitis reaches a remarkable significance in patients at risk of developing infective endocarditis [179]. Figure 11 shows the guidelines for prevention.

Evidence suggests that *Streptococcus mutans* (*S. mutans*) has a role as a major aetiologic agent of dental caries and it is involved in systemic diseases, such as bacterial endocarditis, if it enters the bloodstream through temporary bacteremia. The infection sustained by *S. mutans* is characterized by a high-level synthesis of Interleukin (IL)-1β, a proinflammatory cytokine, that is engaged by the host’s defenses against pathogens. These processes of synthesis, maturation, and secretion were closely adjusted by the activation of the inflammasome, an inflammatory signaling complex. Song et al. examined the signaling mechanism of the *S. mutans*-induced inflammasome pathway at IL-1β secretion, thus securing the basis of the mechanism that can support systemic oral streptococcal infection. Investigators provided novel insight with regards to the innate immune response against *S. mutans* infection. After infection of THP-1 cells with *S. mutans* there was an increase in the inflammasome expression associated with IL-1β secretion through activation of caspase-1, NLRP3, and NLR family CARD domain-containing 4 (NLRC4). Of note is that the *S. mutans*-induced NLRP3 inflammasome activity was mediated by adenosine triphosphate release, potassium depletion, and lysosomal damage [180]. 

*Aggregatibacter actinomycetemcomitans* leads to aggressive periodontitis which denotes the peculiar characteristic of early onset and rapid progression of periodontal destruction. Lee et al. suggest that *A. actinomycetemcomitans* upgrades bacterial internalization by phagocytosis in infected macrophages, by the increase in light chain 3 type II (LC3-II), autophagy-related gene 5/12, and Beclin-1 expression through the Toll-like receptors and extracellular signal-regulated kinase signaling pathways. This process restricted the disproportionate inflammatory response by downregulation of IL-1β and reactive oxygen species (ROS) production. Bacterial internalization through phagocytosis in macrophages could be suppressed through the inhibition of autophagy induced by *A. actinomycetemcomitans*, thereby increasing the production of IL-1β. [181].

### 6.2. Cardiac Device Infection

Infection that occurs in CIEDs is increased and favored by coagulase-negative staphylococci. The trend in infection rates of CDIs, including permanent pacemakers, implantable cardioverter defibrillators, and cardiac resynchronization therapy devices, reaches the incidence of 1 to 10 per 1000 device years (approximately 1 per 1000 device years for pacemakers and 8 to 9 per 1000 device years for complex devices) [182,183,184]. In addition, in the United States, the trend in infection rates of CDIs has exponentially increased due to the increase in implantation rates [128], leading to noticeable higher short- and long-term morbidity and mortality associated with the incremental cost of management [185,186]. IE is due to the onset of risk factors for CDIs that may be patient-, procedure-, or device-related determinants [187]. Patients presenting at great specific risk of infection include those who receive corticosteroid administration, or have heart failure, diabetes mellitus, end-stage kidney disease, chronic obstructive pulmonary disease, malignancy, and previous device infection. Of note is that procedural risk factors suggesting increased development of IE are post-operative hematoma complications (OR: 8.46; 95% CI: 4.01 to 17.86), reintervention due to lead displacement, long procedure times, and implantation of ≥2 leads. In patients who required a revision procedure, IE occurs with a 2- to 5-fold higher risk than the first implantation. The use of antibiotic preoperative prophylaxis administration has been disclosed to give substantial protection against CDI in both RCTs and observational studies. Regarding prophylaxis, aminoglycosides are no longer recommended by the ESC and AHA guidelines for the treatment of methicillin-sensitive or methicillin-resistant *S. aureus* for native valve and cardiac device endocarditis. Although aminoglycosides have represented the class of antibiotics widely used for enterococcus-driven ED, the increasing frequency of resistance, which reaches rates of 25% to 50% in tests reported by recent studies, together with the recognition of potential harm, has driven the ESC Guidelines Committee 2021 to identify ampicillin and ceftriaxone (Class IB recommendation) as treatment of choice for aminoglycoside-resistant *Enterococcus faecalis*, effective as ampicillin/gentamicin, with decreased levels of nephrotoxicity [187,188,189,190,191,192,193,194,195,196].

CDI may develop in various sites, involving the generator pocket, device leads, or endocardial (valvular or non-valvular) surfaces (or any combination of these locations). The main characteristic of the infection located at the level of pocket infections is cellulitis, erythema, wound discharge, and pain. Patients may experience an inchoate or overt erosion of the skin overlying the pocket. On the contrary, in patients who have an infection that involves CIED leads or the endocardial surface (CIED-IE), fevers and rigors occur; frequently, CIED leads or CIED-IE coexists with pocket infections. IE may arise from an infection located at the level of the pocket or it occurs by spreading the infection to the leads via the bloodstream. The diagnosis of CIED-IE is confirmed by echocardiographic evaluation and blood culture results. The TEE procedure is recommended, as it has revealed better sensitivity and specificity than TTE for vegetative detection [197]. A concern may be offered by the presence of sterile clots which are detected in a high percentage of CIED patients without infection. However, these lesions are indistinguishable from infected vegetation [198]. Therefore, in the presence of doubtful cases with a negative or equivocal echocardiographic relationship, the scintigraphy with radiolabeled leukocytes or ^18^FDG–PET/CT scans are of substantial help, thus offering a definitive diagnosis, as shown by some studies that reveal a high sensitivity and specificity for infection [199,200,201]. However, evidence suggests that ^18^18FDG–PET/CT imaging can produce a false negative result for CIED-IE with lead involvement, if patients have received prior antibiotic therapy. Cautela J et al. report that 9 of 13 patients disclosed a false negative scan for CIED-IE, thus reporting a sensitivity of 30.8% [201].

In patients with a confirmed diagnosis of CIED-IE, complete removal of the infected system is indicated because medical therapy alone is associated with an increased risk of relapse and mortality [202,203]. While percutaneous extraction is generally feasible in patients who have received an implantable defibrillator or pacemaker, it is not feasible in recipients of the LVAD system. Treatment with antibiotics alone is associated with a major complication, reaching a rate of 1.9% [204]. Therefore, continued antibiotic therapy is recommended and negative blood culture results should be negative for at least 72 h prior to reimplantation if a new device is crucial [65,196].

Particular attention is paid today to infections caused by *Mycobacterium endocarditis* and *Mycobacterium chimaera* [205,206]. *Mycobacterium endocarditis* is a rare pathogen with the characteristics of rapidly growing mycobacterium. The most frequently used antibiotic therapy is based on the administration of amikacin, ciprofloxacin, and clarithromycin, but poor responses to treatment are often reported. Patients who experience deep-seated infections may require surgery or line withdrawal [205].

*Mycobacterium chimaera* is an opportunistic pathogen included in non-tuberculous mycobacterium and belonging to the *Mycobacterium avium* complex [206]. Generally, the infection leads to a picture of respiratory illness and it is recorded more frequently in individuals presenting with immunodeficiency, such as heart transplant patients or in individuals with underlying respiratory diseases [206]. During the last decade, evidence suggests an increased rate of infection sustained by *Mycobacterium chimaera* following cardiothoracic open-heart surgery procedure. The gate of entry of the causative pathogen is the bioaerosol emitted by the water systems of the contaminated heating–cooling units during cardiopulmonary bypass. The infection is characterized by non-specific symptoms and long latency, so postoperative *Mycobacterium chimaera* infections, if not promptly diagnosed and treated, can become life-threatening [206].

Although revision surgery must be carefully considered on a case-by-case basis, in patients with LVAD or who have received a heart transplant, the indication for revision surgery is recommended. Antibiotic therapy should be guided based on the results of drug sensitivity tests [61,63,192].

However, the difficulty remains biofilm formation. Concerns related to disruption of the biofilm architecture might be more promising with an approach offered by the use of monoclonal antibodies, such as TRL1068, which are currently under evaluation. In an in vivo mouse model, where the formation of a biofilm supported by a methicillin-resistant *S. aureus* infection was found, treatment using a combination of TRL1068 with daptomycin significantly reduced the adherent bacterium count compared to daptomycin alone [207].

It has been suggested that Staphylococci, particularly the CoNS strain, are involved, reaching 60% to 80% of cases, with a critical key role sustained by Fibronectin (fn) and fibrinogen (fg). These molecules are major host proteins present in the extracellular matrix, blood, and coatings on indwelling medical devices. Infections localized on medical devices are strictly dependent on the high capacity of favorable interactions between *Staphylococcus aureus* with these host ligands. The survival and persistence of CoNS *S. aureus* on medical devices may depend on complementary roles offered by fibronectin-binding proteins A and B, as they interact with different conformations of Fn or Fg. The latter may be compact in solution vs. extended on a surface, and are present in different physiological and pathological conditions [208,209].

The interactions are from bacterial adhesins, FnBPA and FnBPB, and host ligands explain the pathogenesis of clumping and adhesion during device infection sustained by the CoNS strain of *S. aureus.* Studies using the combination of seven different strains of *S. aureus* and *Lactococcus lactis*, a Gram-positive surrogate that naturally lacks adhesins to mammalian ligands, suggest that, in the absence of soluble ligands (i.e., fn or fg), both FnBPA and FnBPB are able to interact with adjacent FnBPs of neighboring bacteria to mediate aggregation. With the addition of soluble host ligands, in particular fn, and under shear stress, the aggregation is enhanced. However, FnBPB revealed a greater ability to aggregate than FnBPA, suggesting a distinct role for the two closely related bacterial adhesins. FnBPB and FnBPA have different functional abilities to interact with host ligands in different contexts, such as, for example, the “soluble” or “immobilized” condition [209].

Finally, the decolonization of patients colonized by MRSA is one of the recommended methods for controlling MRSA in hospitals. However, we have a limited choice of agents that can be used. Evidence shows that octenidine dihydrochloride is a relatively new antiseptic and has been used for the decolonization of MRSA in some countries. An evaluation of the literature describing its use highlights only a few observational studies. It is also important to note that all of these studies were based on a small volume of patients and differed in study design. Probably, very variable MRSA decolonization rates between 6 and 75% have been reported. Octenidine appears to be as effective as chlorhexidine for decolonization of MRSA, but reveals fewer adverse effects. However, a better assessment can only be achieved by undertaking large randomized trials incorporating octenidine as a skin disinfectant for the decolonization of MRSA, thus confirming its usefulness in the clinical setting [210]. Intranasal octenidine is an antiseptic alternative to mupirocin that can be used for MRSA decolonization in the prevention of nosocomial transmission. The role of intranasal octenidine was analyzed in a study conducted in three extended-care hospitals from 2015 to 2016. Two hospitals (A and B) administered universal daily chlorhexidine bathing and intranasal octenidine for MRSA colonizers, while in the third hospital (C), no intervention was effectuated. Results suggest that the use of topical intranasal octenidine, coupled with universal daily antiseptic bathing, substantially decreased in extended-care facilities [211].

## 7. Conclusions

Infective endocarditis is only projected to increase with further implantation of devices and transcatheter valves. The need for a vaccine is therefore increasing, given the high-risk nature of this cohort of patients with multiple comorbidities. Early index of suspicion is needed with prompt initiation of treatment and referral to a heart team to ensure good outcomes. Infective endocarditis remains a major burden on healthcare systems, especially in the western world with the ageing population.

## Abbreviations

ADAM	A disintegrin and metalloprotease
ADAMTS13	A disintegrin and metalloprotease with thrombospondin type 1 repeats
Agr	Accessory Gene Regulatory System
Atl	autolysin
CHD	congenital heart disease
CIED	cardiac implantable electronic devices
Clf	cell-bound clumping factor
Coa	coagulase
CoNS	coagulase negative
EC	endothelial cell
Efb1	extracellular fibrinogen binding protein
EPS	extracellular polymeric substances
ESAT-6	Early Secreted Antigenic Target 6 kDa
Fg	fibrinogen
Fnb	fibronectin binding protein
HIV	immunodeficiency virus
Hla	human leukocyte antigen
Hsc70	Heat shock cognate
Hsp60	Heat shock protein
KC	keratinocyte
ICAM	Inter Cellular Adhesion Molecule
IE	Infective endocarditis
IL	interleukine
LAVD	left ventricular assist device
LC3-II	light chain 3 type II
Luk	leukotoxins
MntC	manganese transport protein C
MSCRAMM	microbial surface components recognizing adhesive matrix molecules
NK	natural killers
NLR	Nod-Like receptor
NLRP3	NOD-like receptor family pyrin domain-containing 3
PMN	polymorphonuclear
PVL	proviral load
PWID	persons who inject drugs
*S aureus*	*Staphylococcus aureus*
SpA	Staphylococcal Protein A
SdrD	Serine Aspartate repeat containing protein D
TLR	Toll-like receptor
TNF	tumor necrosis factor
TNFR1	tumor necrosis factor receptor 1.
TSS-1	Toxic Shock Syndrome-1
ULVWF	Ultra Large vWF
United States	US
vWbp	von Willebrand factor-binding protein
vWF	von Willebrand factor
VCAM	vascular cell adhesion molecule
